# A contribution to the fauna of nocturnal macrolepidoptera of La Maddalena and Asinara National Parks, Sardinia

**DOI:** 10.3897/BDJ.13.e171705

**Published:** 2025-11-28

**Authors:** Sara La Cava, Giada Zucco, Michelina Pusceddu, Stephane Knoll, Alberto Satta, Stefano Scalercio

**Affiliations:** 1 Council for Agricultural Research and Economics (CREA), Research Centre for Forestry and Wood, Via Settimio Severo 85, 87036, Rende, Italy Council for Agricultural Research and Economics (CREA), Research Centre for Forestry and Wood, Via Settimio Severo 85, 87036 Rende Italy; 2 Department of Biology, Ecology and Earth Sciences, University of Calabria, Ponte Pietro Bucci, 87036, Rende, Italy Department of Biology, Ecology and Earth Sciences, University of Calabria, Ponte Pietro Bucci, 87036 Rende Italy; 3 Department of AGRARIA, University “Mediterranea” of Reggio Calabria, Feo di Vito, 89122, Reggio Calabria, Italy Department of AGRARIA, University “Mediterranea” of Reggio Calabria, Feo di Vito, 89122 Reggio Calabria Italy; 4 Department of Agricultural Sciences, University of Sassari, Viale Italia 39/A, 07100, Sassari, Italy Department of Agricultural Sciences, University of Sassari, Viale Italia 39/A, 07100 Sassari Italy; 5 NBFC, National Biodiversity Future Center, Piazza Marina 61, 90133, Palermo, Italy NBFC, National Biodiversity Future Center, Piazza Marina 61, 90133 Palermo Italy

**Keywords:** Lepidoptera, Mediterranean Islands, biodiversity

## Abstract

**Background:**

The Mediterranean Basin is recognised as global biodiversity hotspot, with islands playing a crucial role in sustaining high levels of endemism due to their geological complexity, biogeographical history and habitat heterogeneity. Despite this, many Mediterranean islands remain underexplored from a faunistic perspective, particularly regarding nocturnal Lepidoptera.

**New information:**

This study provides new faunistic data on the nocturnal macrolepidoptera of two protected areas in the north of Sardinia: Asinara and La Maddalena islands. A total of 875 specimens were collected, representing 98 species across four families. Of these, 62 species were recorded on the La Maddalena and 78 on the Asinara islands. Notably, nine species are newly reported for La Maddalena, increasing the known macrolepidopteran fauna of the archipelago. Our study provides novel insights especially to the knowledge of the Asinara Island which remains poorly investigated due to its historical restricted access. This study offers a baseline for future ecological and biogeographical research and highlights the need for ongoing faunistic surveys in Mediterranean island systems. Furthermore, this study underscores the biogeographical importance of these islands which host rich moth communities despite their small size.

## Introduction

### Mediterranean Islands as biodiversity hotspots

The Mediterranean Basin is recognised as a global biodiversity hotspot (Cuttelod et al. 2009), characterised by a high concentration of endemic species and a long history of interactions between climatic, geological and anthropogenic factors (Beffa et al. 2016, Perret et al. 2023). Within this region, the Mediterranean islands represent a complex ecological system, differing in shape, size, isolation, geological age and level of human colonisation (Vogiatzakis et al. 2008). These elements contribute to a unique mosaic of ecosystems that promote speciation and the conservation of endemic taxa (Grill et al. 2007). Large islands, such as Sicily, Sardinia, Corsica and Crete, exhibit considerable environmental heterogeneity, including diverse geological substrates - metamorphic, calcareous and volcanic - and a wide range of altitudinal, climatic and biogeographical gradients (Médail and Quézel 1997, Vogiatzakis et al. 2008). These features directly influence the composition and distribution of biological communities, including entomological taxa, which show high levels of endemism, particularly amongst Coleoptera, Plecoptera and Lepidoptera (Blondel and Aronson 1999, Grill et al. 2007). Amongst these islands, Sardinia stands out for its remarkable ecological complexity and biogeographical characteristics. Its landscape is shaped by ancient metamorphic and volcanic formations, as well as by millennia of land-use activities, which have contributed to the formation of a mosaic of semi-natural habitats (Vogiatzakis et al. 2008, Beffa et al. 2016, Perret et al. 2023). This landscape heterogeneity, biogeographical history and its position in the western Mediterranean support a diversified fauna with a rich array of endemic species (Balletto 1995, Grill et al. 2002, Grill et al. 2007), even if, in recent years, it has suffered a significant impoverishment of fauna (Dapporto and Dennis 2009, Stefanescu et al. 2011). According to Karsholt and Razowski (1996), the lepidopteran fauna of Sardinia amounted to about 1500 species. Concerning nocturnal macrolepidoptera, the most updated checklist reported 592 species (Parenzan and Porcelli 2006). This number has been increased during the last decades, thanks to several studies, in some cases describing new endemic species (Hausmann 2011, Fiumi and Flamigni 2013, Fiumi et al. 2013, Schintlmeister and Leipnitz 2014). Furthermore, some focused studies have been carried out in the Island, especially devoted to assessing butterflies and their conservation status (Grill et al. 2002, Grill et al. 2005, Annessi et al. 2025), as well as to faunistic research on macrolepidoptera (Iuli and Zilli 2011, Cao et al. 2012, Bisi and Lupi 2019). Despite smaller islands often being overlooked, they also play a crucial role: these satellite islets, closely linked to the geological and biotic history of larger islands, act as ecological refuges and natural laboratories for evolutionary differentiation (Greuter 1972, Snogerup 1985). In this context, the islands of Asinara and La Maddalena, located in the north-western and north-eastern sectors of Sardinia, are of particular interest. Although limited in size, these islands encompass a rich mosaic of natural and semi-natural habitats, making them an ideal setting for investigating island biodiversity. This is especially true for nocturnal macrolepidoptera, a taxonomic group still poorly studied in Mediterranean island systems, despite its ecological importance and value as a bioindicator (Dar and Jamal 2021). While some recent research has addressed the macrolepidopteran fauna of the Archipelago of La Maddalena and other circumsardinian islands (Mosconi et al. 2018a, Mosconi et al. 2018b, Mosconi et al. 2024), no comparable studies are currently available for Asinara Island, although a general survey of the island's insect fauna has been conducted (Nuvoli et al. 2007). This makes the present paper one of the few contributions to the knowledge on nocturnal Lepidoptera of the island, offering a baseline for future faunistic, ecological and biogeographical investigations.

## Materials and methods

### Study area and sites

The survey was carried out on the islands of Asinara and La Maddalena, located in Sardinia (Italy), both known for hosting national parks; the Asinara National Park and the National Park of the Maddalena Archipelago.

Asinara is a small island located less than two nautical miles from the north-western coast of Sardinia. It covers an area of approximately 52 km² and is predominantly composed of Paleozoic metamorphic rocks, mainly schists and phyllites, with some granitoid intrusions. The island's highest point, Punta della Scomunica, reaches 408 m above sea level.

The vegetation is dominated by Mediterranean maquis and garrigue, with typical species including juniper (*Juniperus* spp.), mastic tree (*Pistacia lentiscus* L.), heather (*Erica arborea* L.), wild olive (Olea europaea L. var. sylvestris Brot.), rockroses (*Cistus* spp.) and rosemary (*Salvia rosmarinus* Spenn.). Halophytic and psammophilous plant communities are found along the coasts, while more sheltered and elevated areas host remnants of holm oak (*Quercus ilex* L.) woodlands (Bocchieri 1988).

The climate of Asinara is Mediterranean with strong oceanic influence, marked by hot, dry summers and mild, wetter winters. According to the bioclimatic classification of Canu et al. (2015), the island falls within the Oceanic Pluviseasonal Mediterranean bioclimate, upper thermo-Mediterranean phytoclimatic belt.

La Maddalena is the main island of the Archipelago bearing the same name, situated in north-eastern Sardinia within the Strait of Bonifacio, which separates Sardinia from Corsica. It covers an area of approximately 51 km² and is predominantly composed of granitoid rocks that formed around 300 million years ago. The island’s maximum elevation, Monte Guardia Vecchia, reaches 146 m above sea level.

The island’s vegetation is dominated by Mediterranean maquis, consisting primarily of juniper *(Juniperus* spp.), myrtle (*Myrtus communis* L.), strawberry tree (*Arbutus unedo* L.), mastic tree (*Pistacia lentiscus*), mock privet (*Phillyrea* spp.), tree heath (*Erica arborea*), rockrose (*Cistus* spp.), spurge (*Euphorbia* spp.) and rosemary (*Salvia rosmarinus*), along with patches of garrigue and halophytic vegetation along the coastal zones (Biondi and Bagella 2005).

The climate is typically Mediterranean, with hot, dry summers and mild, wetter winters, strongly influenced by prevailing winds, especially the westerly (Ponente). According to the bioclimatic classification of Canu et al. (2015), the area falls within the Oceanic Pluviseasonal Mediterranean bioclimate, upper thermomediterranean thermotype and lower dry ombrotype.

### Moths sampling method

Lepidoptera samples were collected monthly from April to November 2024, for a total of eight sampling sessions in each National Park. Sampling events were scheduled at approximately four-week intervals, although this frequency occasionally varied due to weather constraints as samples could only be collected on nights with conditions favourable to Lepidoptera activity (La Cava et al. 2024). Bucket traps equipped with 150 UV LEDs (total output: 15 watts) powered by external batteries were used for collection (Infusino et al. 2017). Traps were positioned along existing pollinator monitoring transects within each National Park (Lezzeri et al. 2024). In Asinara National Park, the trap was placed near Fornelli (40.99873°N, 8.24345°E) at an altitude of 35 m, while in the National Park of the Maddalena Archipelago near Cala Spalmatore (41.247402°N, 9.423127°E) at an altitude of 44 m. The traps in both locations were placed in a habitat that is very common in Sardinia, namely semi-open Mediterranean maquis, dominated by various shrubs, areas with herbaceous plant communities and bare rock. Each trap was mounted on a metal pole with a bracket, allowing installation at approximately 1.3 m above ground level (La Cava et al. 2024). Inside each trap, a 200 ml vial with a perforated cap contained a hemp cord, which served as a slow-release dispenser of ethyl acetate used to kill the captured insects (Scalercio et al. 2022). Traps were activated at dusk and deactivated at dawn, after which the contents were collected. The captured macrolepidoptera were subsequently prepared and pinned and then identified by specialist taxonomists.

### Specimens identification

Moth identification was carried out using available literature, such as The Geometrid Moths of Europe and Noctuidae Europaea (Fibiger and Hacker 2011, Hausmann et al. 2023) and iconographic sources as well as through genitalia dissection in the case of cryptic species or when specimens were too worn to be reliably identified, based on wing patterns.

### Results

A total of 875 specimens were collected representing 98 species across four families. Of these, 255 specimens belonging to 62 species were recorded from La Maddalena and 620 specimens belonging to 78 species from Asinara Island. The most represented family was Geometridae, with 41 species, followed by Noctuidae (38 species), Erebidae (18 species) and Euteliidae (1 species). The supplementary file (Suppl. material 1) contains all species collected during this study, including all new records for both Asinara and La Maddalena National Parks, following the taxonomic arrangement adopted by Lepiforum (lepiforum.org). In the next section, only moth species newly recorded from La Maddalena National Park are reported.

## Data resources

All nocturnal macrolepidoptera species occurrences in La Maddalena and Asinara National Parks are also deposited at https://doi.org/10.15468/g7fybj

## Checklists

### Moth species newly recorded from La Maddalena National Park

#### Pachycnemia tibiaria benesignata

Herbulot, 1944

711F2EF3-AED2-5B79-A866-5FFC95232D96

##### Materials

- **Type status:** Other material. **Occurrence:** recordedBy: S. Knoll; individualCount: 6; lifeStage: adult; occurrenceID: 9ED70146-AB86-5A53-8256-BEF47E07070C; **Location:** island: La Maddalena; country: Italy; countryCode: IT; stateProvince: Sassari; county: Sardinia; municipality: La Maddalena; locality: La Maddalena; decimalLatitude: 41.247402; decimalLongitude: 9.423127; geodeticDatum: WGS84; **Identification:** identifiedBy: S. Scalercio and S. La Cava; **Event:** eventDate: 15/10/2024

#### Ekboarmia atlanticaria

(Staudinger, 1859)

676360E5-34E4-5A37-8AF3-1F915E2CA20E

##### Materials

- **Type status:** Other material. **Occurrence:** recordedBy: S. Knoll; individualCount: 1; lifeStage: adult; occurrenceID: 60ABBA25-71D9-58B6-9189-394834306FE7; **Location:** island: La Maddalena; country: Italy; countryCode: IT; stateProvince: Sassari; county: Sardinia; municipality: La Maddalena; locality: La Maddalena; decimalLatitude: 41.247402; decimalLongitude: 9.423127; geodeticDatum: WGS84; **Identification:** identifiedBy: S. Scalercio and S. La Cava; **Event:** eventDate: 28/08/2024
- **Type status:** Other material. **Occurrence:** recordedBy: S. Knoll; individualCount: 1; lifeStage: adult; occurrenceID: 3CD16C4A-5945-58D1-A934-3493AC8C7B9D; **Location:** island: La Maddalena; country: Italy; countryCode: IT; stateProvince: Sassari; county: Sardinia; municipality: La Maddalena; locality: La Maddalena; decimalLatitude: 41.247402; decimalLongitude: 9.423127; geodeticDatum: WGS84; **Identification:** identifiedBy: S. Scalercio and S. La Cava; **Event:** eventDate: 15/10/2024

#### Eupithecia ultimaria

Boisduval, 1840

21B63CCB-D22D-5153-AEF7-ACAD9B9F92D7

##### Materials

- **Type status:** Other material. **Occurrence:** recordedBy: S. Knoll; individualCount: 1; lifeStage: adult; occurrenceID: 466908DF-8905-5061-8726-432002625107; **Location:** island: La Maddalena; country: Italy; countryCode: IT; stateProvince: Sassari; county: Sardinia; municipality: La Maddalena; locality: La Maddalena; decimalLatitude: 41.247402; decimalLongitude: 9.423127; geodeticDatum: WGS84; **Identification:** identifiedBy: S. Scalercio and S. La Cava; **Event:** eventDate: 28/08/2024

#### Agrotis lata

Treitschke, 1835

CE6802BD-EAC7-546A-B8D2-30776F467F34

##### Materials

- **Type status:** Other material. **Occurrence:** recordedBy: S. Knoll; individualCount: 3; lifeStage: adult; occurrenceID: 95F501D9-FD17-5681-AC11-54D00268CF0A; **Location:** island: La Maddalena; country: Italy; countryCode: IT; stateProvince: Sassari; county: Sardinia; municipality: La Maddalena; locality: La Maddalena; decimalLatitude: 41.247402; decimalLongitude: 9.423127; geodeticDatum: WGS84; **Identification:** identifiedBy: S. Scalercio and S. La Cava; **Event:** eventDate: 20/09/2024

#### Parascotia nisseni

Turati, 1905

9D2BCCCD-8256-5123-8C63-37DFAB6E6A70

##### Materials

- **Type status:** Other material. **Occurrence:** recordedBy: S. Knoll; individualCount: 1; lifeStage: adult; occurrenceID: 46ED72CB-E60D-5C18-BC7B-746C84ECA3F5; **Location:** island: La Maddalena; country: Italy; countryCode: IT; stateProvince: Sassari; county: Sardinia; municipality: La Maddalena; locality: La Maddalena; decimalLatitude: 41.247402; decimalLongitude: 9.423127; geodeticDatum: WGS84; **Identification:** identifiedBy: S. Scalercio and S. La Cava; **Event:** eventDate: 20/09/2024

#### Grammodes bifasciata

(Petagna, 1786)

5A0A162C-7C96-56AA-BC67-0B298C47772D

##### Materials

- **Type status:** Other material. **Occurrence:** recordedBy: S. Knoll; individualCount: 1; lifeStage: adult; occurrenceID: AB09D212-7521-53F2-B62D-9E3A2F554F6E; **Location:** island: La Maddalena; country: Italy; countryCode: IT; stateProvince: Sassari; county: Sardinia; municipality: La Maddalena; locality: La Maddalena; decimalLatitude: 41.247402; decimalLongitude: 9.423127; geodeticDatum: WGS84; **Identification:** identifiedBy: S. Scalercio and S. La Cava; **Event:** eventDate: 20/09/2024

#### Coenotephria ablutaria

(Boisduval, 1840)

060F622F-FE24-5B50-A9D6-48E1F71977D7

##### Materials

- **Type status:** Other material. **Occurrence:** recordedBy: S. Knoll; individualCount: 13; lifeStage: adult; occurrenceID: 7CA85A8F-C6D5-5A2F-872B-82734D6C9CB6; **Location:** island: La Maddalena; country: Italy; countryCode: IT; stateProvince: Sassari; county: Sardinia; municipality: La Maddalena; locality: La Maddalena; decimalLatitude: 41.247402; decimalLongitude: 9.423127; geodeticDatum: WGS84; **Identification:** identifiedBy: S. Scalercio and S. La Cava; **Event:** eventDate: 15/10/2024

#### Euproctis chrysorrhoea

(Linnaeus, 1758)

118B8044-E793-57C5-8949-4ADDB51B1293

##### Materials

- **Type status:** Other material. **Occurrence:** recordedBy: S. Knoll; individualCount: 2; lifeStage: adult; occurrenceID: 4693C7C5-0E60-5AE3-A0DC-27EC5844B432; **Location:** island: La Maddalena; country: Italy; countryCode: IT; stateProvince: Sassari; county: Sardinia; municipality: La Maddalena; locality: La Maddalena; decimalLatitude: 41.247402; decimalLongitude: 9.423127; geodeticDatum: WGS84; **Identification:** identifiedBy: S. Scalercio and S. La Cava; **Event:** eventDate: 20/06/2024

#### Cryphia algae

(Fabricius, 1775)

3188B82F-16C5-557F-9C7D-5D12B8A17F8D

##### Materials

- **Type status:** Other material. **Occurrence:** recordedBy: S. Knoll; individualCount: 3; lifeStage: adult; occurrenceID: 3C747FA0-C69E-533E-8CB0-227385C6F842; **Location:** island: La Maddalena; country: Italy; countryCode: IT; stateProvince: Sassari; county: Sardinia; municipality: La Maddalena; locality: La Maddalena; decimalLatitude: 41.247402; decimalLongitude: 9.423127; geodeticDatum: WGS84; **Identification:** identifiedBy: S. Scalercio & S. La Cava; **Event:** eventDate: 28/08/2024
- **Type status:** Other material. **Occurrence:** recordedBy: S. Knoll; individualCount: 1; lifeStage: adult; occurrenceID: 4D9E0132-E24E-500F-A483-6FD3E5BC1D0D; **Location:** island: La Maddalena; country: Italy; countryCode: IT; stateProvince: Sassari; county: Sardinia; municipality: La Maddalena; locality: La Maddalena; decimalLatitude: 41.247402; decimalLongitude: 9.423127; geodeticDatum: WGS84; **Identification:** identifiedBy: S. Scalercio & S. La Cava; **Event:** eventDate: 15/10/2024

## Discussion

This paper presents new data on the macrolepidoptera of the La Maddalena Archipelago National Park and the Asinara National Park. In addition to the species recorded by Mosconi et al. (2024), we report nine species as new for the island of La Maddalena. Two species are absent from the Italian mainland, namely *Pachycnemia tibiaria benesignata*, also recorded in the Tuscan Archipelago and Corsica (Parenzan and Porcelli 2006) and *Ekboarmia atlanticaria*, known from Corsica, North Africa, the Iberian Peninsula and the Balearic Islands (Redondo et al. 2009). *Pachycnemia tibiaria benesignata* prefers Mediterranean maquis habitats with scattered shrubs and trees (Skou and Sihvonen 2014) and occurs from sea level up to 1,400 m. Its larvae are oligophagous, feeding mainly on *Calluna vulgaris* (L.) Hull., Erica scoparia L. subsp. scoparia and *E. arborea*; *Ekboarmia atlanticaria*, by contrast, inhabits a variety of habitats where its host plants occur, from coastal dunes to inland areas up to about 300 m (Skou and Sihvonen 2014). The species is considered monophagous on *Juniperus phoenicea* L.; however, according to Conti et al. (2005), this juniper species has been erroneously reported for Sardinia. It is, therefore, possible that the larvae of *E. atlanticaria* feed on other *Juniperus* species present on the island, thus being monophagous on the genus rather than on a single species. Seven additional species recorded from La Maddalena are also known from continental Italy. Two of them have a restricted range on the Italian mainland: *Eupithecia ultimaria*, with a Mediterranean–Turanian distribution (Mironov 2003, Rijllo et al. 2024) and *Agrotis lata*, an Atlanto-Mediterranean species (Fibiger 1990). *Eupithecia ultimaria* typically occurs in humid or riparian habitats, inhabiting wetland margins or areas with *Tamarix* shrubs, its larval host plants (Mironov 2003, Rijllo et al. 2024). *Agrotis lata* inhabits open areas with scattered vegetation (Fibiger 1990), but has also been found within cultivated areas of south Italy (Zucco et al. 2024); its larvae feed on grasses (Fibiger 1990). Two further species are widespread in warm habitats: *Parascotia nisseni*, occurring in North Africa and, in Europe, in the Iberian Peninsula, Provence, southern Italy and Sicily (Fibiger et al. 2010); and *Grammodes bifasciata*, widespread in southern Europe, including the Balearic Islands (Goater et al. 2003). *Parascotia nisseni* is associated with Mediterranean sclerophyllous woodlands and its larvae feed on fungi and lichens growing on wood and soil (Fibiger et al. 2010). *Grammodes bifasciata* occurs in hot, dry areas, both cultivated and uncultivated, especially near the Mediterranean coast; its larvae feed on various shrubs such as *Rubus* spp. and *Cistus* spp., typical of the Mediterranean maquis (Goater et al. 2003). Three more species, namely *Coenotephria ablutaria*, *Euproctis chrysorrhoea* and *Cryphia algae*, are also common in mountain habitats (Piccini et al. 2023, La Cava et al. 2024). *Coenotephria ablutaria*, widely distributed in the eastern Mediterranean Basin and typically restricted to habitats such as maquis and scattered oak forests (Hausmann and Viidalepp 2012), whose larvae are monophagous on *Galium* spp; *Euproctis chrysorrhoea*, widespread throughout Europe and also recorded in Russia, Iran and Turkey (De Freina and Witt 1987), inhabiting riparian forests, orchards and gardens from lowlands to mountains, whose larvae feed mainly on *Quercus*, *Fagus*, *Ulmus*, *Rubus* and *Crataegus* species (Fibiger et al. 2011); *Cryphia algae*, distributed across Europe and the Near East, inhabiting xeric to mesophilous woodlands, shrublands, orchards, parks and gardens, whose larvae feed on lichens growing on trunks of *Quercus* and *Populus* species, as well as on fruit tree branches and dead wood (Fibiger et al. 2009).

With the establishment of the National Park, scientific interest in the fauna of Asinara Island has grown. However, its long history of isolation due to the presence of healthcare and penitentiary facilities has long obstructed detailed naturalistic studies. For this reason, data on the island’s entomofauna remain fragmented and poorly explored (Nuvoli et al. 2007). In this context, the present survey represents the most substantial contribution to data regarding nocturnal Lepidoptera. Previously, only 26 Lepidoptera species had been recorded from the island, with just one belonging to any of the families included in our study (Nuvoli et al. 2007). As a result, all the species recorded here are new for the Asinara National Park. Amongst the taxa sampled, the presence of the Sardinian endemic subspecies, *Hadena sancta protai* Berio, 1978, is particularly noteworthy. This species inhabits steppe and semi-desert localities vegetated with *Silene* spp., its larval host plants (Hacker et al. 2002). Additionally, seven species are Sardinian-Corsican endemics: *Sardocyrnia bastelicaria* (Bellier, 1862), a xerothermophilous species inhabiting various open habitats with scattered trees and bushes from sea level up to 1,400 m, whose larvae are oligophagous on plants, such as *Erica* spp. and *Santolina corsica* Jord. & Fourr., an Asteraceae endemic to Sardinia and Corsica (Conti et al. 2005, Müller et al. 2019); *Isturgia assimilaria* (Rambur, 1833), which occurs in a variety of habitats ranging from dry to moist open land with scattered trees and bushes and also in denser woodlands, whose larvae are monophagous on *Genista* spp. (Skou and Sihvonen 2014); *Tephronia nuragica* Fiumi, Flamigni, Zilli & Hausmann, 2013, a xerophilous species found from sea level up to 1,100 m in forest fringes, Mediterranean shrublands and pine forests with small clearings, whose larvae probably feed on lichens (Müller et al. 2019); *Scotopterix proximaria* (Rambur, 1833), preferring dry mountain grasslands, rocky slopes, forest fringes and clearings, whose larvae are oligophagous on Fabaceae (Hausmann and Viidalepp 2012); *Xanthorhoe sardisjuncta* Hausmann, 2011, probably inhabiting lowland, whose larvae are oligophagous on Brassicaceae (Hausmann and Viidalepp 2012); *Horisme predotai* Bytinski-Salz, 1937, xerothermophilous species, inhabiting scattered forests and forest clearings, whose larvae are probably oligophagous on *Clematis* spp. (Hausmann and Viidalepp 2012); and *Lerautia bifasciata* (Rambur, 1832), a xerothermophilous species occurring in hilly and meso-montane dry grasslands and karst areas, whose larvae are polyphagous on various low plants (Fibiger et al. 2011). Two species and one subspecies are endemic to Sardinia, Corsica and the Tuscan Archipelago: *Charissa corsica*, a xerothermophilous species preferring open, bushy and rocky habitats, such as rocky valleys, oak and gorse heaths and bushy wastelands, from sea level up to 1,200 m (Müller et al. 2019); *Xestia jordani* (Turati, 1912), which inhabits mountain habitats from 800 to 1,200 m (Fibiger 1993); and *Pachycnemia tibiaria benesignata*. Other noteworthy findings include species that, although present in various parts of Europe, Asia or Africa, are, in Italy, restricted to islands: *Caradrina fuscicornis* Rambur, 1832, known from Corsica, Sardinia and the mainland of South France along the coast (Fibiger and Hacker 2007); *Ekboarmia atlanticaria* know only from Sardinia; *Nebula ibericata* (Staudinger, 1871), xerothermophilous species, preferring open habitats of the lowland and foothills, whose larvae are oligophagous on Rubiaceae (Hausmann and Viidalepp 2012); and *Aporophyla chioleuca* (Herrich-Schäffer, [1850]), found in Sardinia and Sicily inhabiting xerophilous scrub and clearings on gypsum or calcareous substrates, but also on acidic sandy soils at low and medium altitudes up to 800 m, both near the coast and inland. Its larvae feed on various herbaceous plants (Ronkay et al. 2001); *Miniotype spinosa* (Chrétien, 1911), recorded in Sardinia, Sicily and the Tuscan archipelago, inhabits seasonally dry, warm Mediterranean grasslands, scrubs, verges, reedbeds, gardens and other open natural or modified habitats from sea level up to about 800 m, usually not far from the coast. Its food plants are diverse, including the leaves of small trees, shrubs and herbaceous plants; larvae have been recorded on *Rosa* sp., *Arbutus unedo* L., *Hedera helix* L., *Melissa officinalis* L. and *Viola odorata* L. (Ronkay et al. 2001); *Ocneria atlantica* (Rambur, 1837), present in Sardinia, the Tuscan, Sardinian and Sicilian Archipelagos, inhabits garrigue-like scrub and coastal areas (Wagner 2005) and its larvae feed on *Pistacia lentiscus* and *Rhus* spp. (Fibiger et al. 2011).

The ecological traits of the species newly recorded from La Maddalena and Asinara highlight the presence of a complex mosaic of habitats, ranging from Mediterranean maquis and coastal shrublands to more humid and open environments. This habitat heterogeneity supports both xerothermophilous and mesophilous taxa, reflecting the ecological diversity and conservation value of these islands for lepidopteran fauna. The co-existence of widespread species, insular endemics and taxa with limited Mediterranean distributions underlines their biogeographic significance within the Mediterranean Basin. Overall, these findings emphasise the importance of Sardinia’s satellite islands as key sites for understanding Lepidoptera diversity and distribution. Continued faunistic research, particularly on underexplored groups such as microlepidoptera, is likely to reveal additional species and improve our understanding of insular moth communities and their conservation needs.

## Supplementary Material

XML Treatment for Pachycnemia tibiaria benesignata

XML Treatment for Ekboarmia atlanticaria

XML Treatment for Eupithecia ultimaria

XML Treatment for Agrotis lata

XML Treatment for Parascotia nisseni

XML Treatment for Grammodes bifasciata

XML Treatment for Coenotephria ablutaria

XML Treatment for Euproctis chrysorrhoea

XML Treatment for Cryphia algae

F00F92A4-8BF6-525A-B213-52B544C6E375 10.3897/BDJ.13.e171705.suppl1 Supplementary material 1 Species occurrence Data type Occurrences Brief description Nocturnal macrolepidoptera species occurrence in La Maddalena and Asinara National Parks. File: oo_1411481.xlsx https://binary.pensoft.net/file/1411481 La Cava S, Zucco G, Pusceddu M, Knoll S, Satta A, Scalercio S
